# What is the most appropriate method for the measurement of the range of motion in the lumbar spine and how does surgical fixation affect the range of movement of the lumbar spine in adolescent idiopathic scoliosis? A systematic review protocol

**DOI:** 10.1186/s13643-022-02077-1

**Published:** 2022-09-30

**Authors:** Laura Hartley, Mattia Zappalà, Uzo Ehiogu, Nicola R. Heneghan, Adrian Gardner

**Affiliations:** 1 The Royal Orthopaedic Hospital NHS Foundation Trust, The Woodlands, Bristol Road South, Northfield, Birmingham, B31 2AP UK; 2 School of Sport, Exercise and Rehabilitation Sciences, University of Birmingham, Birmingham, UK; 3 Institute of Clinical Sciences, University of Birmingham, Birmingham, UK

**Keywords:** Adolescent idiopathic scoliosis, Scoliosis, Lumbar motion, Range of motion, Spinal motion, Function, Surgery, Surgical intervention, Instrumentation, Fusion, Fixation

## Abstract

**Introduction:**

Adolescent idiopathic scoliosis (AIS) is a three-dimensional rotational change in the normal shape of the spine which affects children aged 10 to 18 years. Both the condition and its management can have significant impact on functional ability. Currently, expected restriction in spinal motion is experience based, rather than evidence based, and discussions to inform patient expectations pre-operatively can be difficult. The aim of this review is to evaluate the evidence pertaining to measurement of spinal motion and whether this is altered following surgery, dependent on the anatomical level of surgical fixation in AIS.

**Methods/analysis:**

This protocol is reported in line with both PRISMA-P and informed by the COSMIN methodology. Electronic databases will be searched using a two-stage search strategy. The first stage will identify and evaluate the methods used to assess spinal motion. The second stage aims to evaluate the change in spinal motion using these methods based on anatomical level of fixation following surgery along with the measurement properties of those methods, to include the validity, reliability and responsiveness of the methods. Two reviewers will independently screen the search results against eligibility criteria, extract the data and assess the quality of the included studies. Any disputes between the reviewers will be resolved with a third independent reviewer. Data may be pooled where possible; however, this is not expected. The overall strength of the body of evidence will then be assessed using the Grading of Recommendations, Assessment, Development and Evaluation (GRADE) approach.

**Patient and public involvement:**

Patients and members of the public will not be consulted in the production of this review, although the review was conceived based on the experiences of the authors when managing this patient population and a need to address patient expectations in pre-operative planning.

**Ethics, dissemination and data availability:**

No ethical approval required. The final review will be submitted to peer-reviewed journals for publication and disseminated publicly. The datasets used and/or analysed in this review will be available from the corresponding author on reasonable request.

**Systematic review registration:**

PROSPERO registration number.

CRD42021282264.

**Supplementary Information:**

The online version contains supplementary material available at 10.1186/s13643-022-02077-1.

## Strengths and limitations

- A two-stage search strategy will be used to identify current methods of assessing spinal motion and then, using the methods identified in stage 1, identify evidence regarding how spinal motion is affected with different levels of surgical fixation in AIS.
- This protocol has been designed in line with COSMIN methodology.
- Two independent reviewers have been used to extract data, assess quality and perform the analysis.
- Excluding studies published in languages other than English may result in some relevant studies being excluded.

## Introduction

Scoliosis is defined as an abnormal lateral curvature of the spine with rotational deformity and a Cobb angle greater than 10 degrees in the coronal plane [1]. In the absence of an identifiable underlying cause and when diagnosed between the ages of 10 and 18 years, it is known as adolescent idiopathic scoliosis (AIS) [2]. AIS is the most common form of scoliosis, affecting up to 4% of teenagers with a roughly equal sex distribution [1]. ‘Idiopathic’ refers to the unclear aetiology of this condition however, hormonal imbalance, asymmetric growth and muscle imbalance are thought to be contributory [3]. Up to 30% of those affected have a positive family history indicating a genetic component [3]. Generally, AIS curves will progress during the rapid growth that accompanies puberty and most curves will eventually slow their progression by the time skeletal maturity is reached [1–4]. Some curves continue to progress into adulthood [1].

Whilst untreated AIS does not increase mortality rates [4], when compared with those without scoliosis, most teenagers with AIS function at or near normal levels [4]. Severe curves may impair functional ability and cause pain [4, 5]. In those affected, the potential caregiver and financial burden can be significant and, therefore, the economic impact of AIS is greater than in healthy age-matched peers [5].

The management of AIS primarily aims to prevent progression through minimising deformity alongside maximising function [6]. Patients with a Cobb angle of 25–30° prior to skeletal maturity, or less than 45° after, may be managed with radiographic surveillance [7]. Similarly, bracing has been shown to be effective in reducing the need for surgical intervention [8]. Surgical intervention aims to correct the deformity, prevent future progression and improve functional ability [6]. It should be considered for those with curves greater than 45° prior to skeletal maturity and those with progression beyond 45° after skeletal maturity is achieved [9].

Many methods exist for determining the anatomical inferior level at which instrumentation should extend. The decision takes into account various factors including curve type, Cobb angle, pre-operative mobility (a systematic review of which method of assessment is most reliable and accurate is underway by another group [10]) and the likely post-operative restriction in movement [11–13]. Restriction in spinal motion is a recognised and expected outcome of corrective surgery for deformity in AIS [4]. When discussing surgical intervention, information regarding the reduction in spinal motion post-operatively is usually experience-based rather than evidence based. It can be difficult to predict how much movement may be lost and therefore what post-operative activities an individual may experience difficulty with [11]. Recent studies have highlighted that the restriction in motion post-operatively is greater with increasing overall number of levels of fixation [12, 13] and with instrumentation that extends to and caudal to L3/4 [12–14]. Over time, abnormal or increased segmental motion in the adjacent unfused spinal levels post fixation, thought to lead to degeneration, and may be responsible for ongoing pain [14–18]. Avoidance of instrumenting the inferior lumbar spine has been recommended where possible to reduce the risk of post-operative pain and preserve as much spinal motion as possible [19–22].

Current methods for assessing the post-operative spinal shape [23] are useful to assess the level of correction achieved and detect complications such as metalwork failure; however, they are limited by their inability to quantify parameters related to spinal motion and have been cited to be unreliable, insensitive and non-specific, especially in the context of ongoing pain [24–27]. Alternative strategies exist, including the use of skin based motion trackers [17] and fingertip-to-floor distance [14]; however, these have also been found to be inaccurate and limited by their inability to pinpoint precise anatomical areas of abnormal motion within the spine. Quantitative fluoroscopy (QF) has shown promise in the assessment and identification of potential therapeutic targets in chronic, non-specific lower back pain (CNSLBP) [24–28]. Several studies have been carried out assessing the validity of QF both in healthy controls and individuals with CNSLBP, where it has been shown to be both reliable and valid [24, 29–31]. QF also has the added benefit of lower overall radiation exposure compared to lumbar spine radiography [32]. However, QF has not yet been investigated in AIS. To our knowledge, no studies exist comparing pre-operative spinal motion directly with post-operative spinal motion in AIS and whilst studies exist looking at the level of function post-operatively based on instrumentation levels, the results are mixed [14, 16, 19].

## Aims

Our aims with this study are twofold. Firstly is to collate and evaluate current methods of assessing spinal motion in AIS. Secondly is to compare these identified methods for measuring pre- to post-operative spinal motion based on the level of instrumentation along with the measurement properties of these methods. Combined, this data will allow the assessment of methods for the measurement, and any post-operative loss of movement.

## Design and methods

This protocol has been designed according to the Consensus-based Standards for the Selection of Health Measurement Instruments (COSMIN) methodology for systematic reviews [33] and is reported in line with Preferred Reporting Items for Systematic Review and Meta-Analysis Protocol (PRISMA-P) [34]. The design and methods have been informed through a collaboration between experts in physiotherapy, rehabilitation and scoliosis management. It has also been registered in the International Prospective Register of Systematic Reviews (PROSPERO ID – CRD42021282264).

The proposed methodology has a two-stage approach—in stage 1, scoping searches will be conducted to identify both historic and contemporary methods of spinal motion assessment in AIS. In stage 2, searches will be conducted for studies that evaluate the effects of surgery on spinal motion based on the anatomical levels of instrumentation.

### Stage 1: Identifying and evaluating the methods used to assess spinal motion

#### Eligibility criteria

##### Population

Studies assessing spinal motion in the age group defined as AIS [2] (i.e. aged 10 to 18 years inclusive) will be included.

##### Outcome

Any study that includes assessment of spinal motion will be included. No restrictions will be applied on the method of assessment of spinal motion.

##### Study design

Any study design reporting quantitative data will be included, e.g. randomised clinical trials, cohort, observational and case studies. There will be no limitation placed on geographical location. Studies published in languages other than English will be excluded.

#### Search strategy

The search strategy has been developed following scoping searches and discussions with experts (subject specific, methodological and a medical librarian). It will involve systematic searches of electronic databases with structured search blocks. The search will be completed by one reviewer (LH). Stage 1 search blocks will contain terms relevant to the population of interest (patients defined as having AIS [2]) and the outcome of interest (spinal motion, any parameter, measured by any method). Unformatted terms may also be used-these are free text terms that require appropriate formatting, prefixing and/or suffixing for searching the relevant database. An example of the search strategy used can be found in Additional file 1.

The title and abstracts of generated studies will be screened independently by two reviewers (LH and MZ). Studies will be categorised as relevant, irrelevant or unsure. Any studies considered irrelevant based on title and abstract by both reviewers will be excluded at this stage. Where both reviewers are unsure or disagree, a third arbitrating reviewer (AG) will make the final decision. Studies deemed relevant to the study question in stage 1 based on abstract and title will proceed to data extraction; the full text articles will be reviewed and categorised into relevant, irrelevant and unsure. For relevant studies, the method used to assess spinal motion will be recorded along with the outcomes. Irrelevant studies will be excluded. Studies where the reviewers are unsure will be reviewed by a third arbitrating reviewer (AG) and, if deemed relevant, will proceed to data extraction. This first stage is expected to yield a list of methods used to evaluate spinal motion, along with outcomes, merits and disadvantages of each method.

### Stage 2: Evaluating change in spinal motion post-operatively dependent on the anatomical levels of instrumentation

#### Eligibility criteria

##### Population

Patients aged 10 to 18 years, diagnosed with AIS, managed with surgical intervention of any type, will be included. Animal studies will not be considered.

##### Outcome

Any study that includes the assessment of post-operative spinal motion based on level of instrumentation will be included. No restrictions will be applied on method of assessment of post-operative spinal motion or to the mode of instrumentation used.

##### Study design

The study design is as described in the stage 1 inclusion criteria.

#### Information sources

The search strategy for stage 2 will involve systematic searches of electronic databases, trial registries, grey literature and experts in the field. An electronic search will be performed through PubMed, Scopus, Web of Science, Cochrane database, EMBASE, MedLine and Ovid bibliographic databases from inception to the date of the last search. Other sources will be searched for grey literature, ProQuest for dissertations and meeting abstracts through Scopus, Web of Science and pertinent websites. Reference lists of relevant studies and systematic reviews will be searched as well.

#### Search strategy

Searches of the electronic databases will be conducted in a similar manner to stage 1 using structured search blocks in order to identify studies that measure changes in spinal motion following surgery using the methods identified in stage 1. The search will be conducted by one reviewer (LH). To identify appropriate key words, in addition to Medical Subject Headings (MeSH terms), popular and commonly used phrases stated in relation to the literature will be used. The search strategy will be developed in MedLine, then the same search strategy applied to the other databases. The search will be conducted using terms relevant to the population of interest (patients with AIS between 10 and 18 years old inclusive) and method of assessing spinal motion identified in stage 1 with the limits detailed above applied. An example of the search strategy used can be found in Additional file 2.

#### Selection process

Two authors will independently perform the initial title and abstract screening (LH and MZ). The studies will be organised into three groups-relevant, irrelevant and unsure. Studies categorised as ‘irrelevant’ based on title and abstract by both reviewers will be excluded from the study. Where studies are categorised as ‘unsure’ by both reviewers or where there is a disagreement, there will be a third review by an arbitrator (AG). Once a list of studies to be considered has been generated, searching for full text articles will take place. Both reviewers will then review the full text of the eligible studies and organise them into ‘relevant’, ‘irrelevant’ and ‘unsure’ groups. The two lists will then be compared and any non-conformities (particularly where both reviewers are unsure) discussed with the third arbitrating reviewer (AG) in the same manner as in stage 1. Relevant studies will proceed to critical appraisal and data extraction. Irrelevant studies will be excluded. This will yield a list of studies relevant to the study question to be included in the review.

#### Critical appraisal of studies and risk of bias

Critical appraisal of the studies will be undertaken using the COSMIN Risk of Bias tool checklists relevant to the individual study types to assess the methodological quality of each article [34]. The full texts will be appraised by two reviewers independently (LH and MZ). Studies of sufficient quality will then proceed to data extraction and synthesis.

#### Data management

Eligible search records will be imported into Mendeley Reference Management software (London, UK). If required data is missing from the full text papers, or are incomplete or unclear, enquiries will be sent to the authors.

#### Data items

From each paper, the following data will be extracted.

**Table:** 

**Study and participant details**	Reference, year of publication, geographical location of study, age, sex, sample size, curve type, curve severity and pattern, surgical intervention including but not limited to type of instrumentation, number of levels fused, most superior (upper instrumented vertebra or UIV) or inferior (lowest instrumented vertebra or LIV), complications
**Method of post-operative spinal motion measurement**	Name of method, outcomes obtained (including but not limited to rotational stability, motion sharing inequality and motion sharing variance), assessment of method (including but not limited to measures of validity, reliability, sensitivity and specificity)
**Method properties**	*Validity* (including type of validity, descriptive statistics, comparator/predictor outcome, hypothesis, missing value, confidence intervals, sample size and validation results) *Reliability* (including type, descriptive statistics, time intervals, reliability coefficients, measurement errors, number of repeated measurements and sample size) *Responsiveness* (including method of testing, whether hypothesis tested, distribution or anchor based, follow-up length, severity of curve prior to surgery, aetiology if available, intervention modality) *Interpretability* (including distribution of scores within the population studied) *Feasibility* (including type and ease of surgical intervention, patient comprehension, cost of intervention, required equipment if stated, availability in different settings, applicability of study sample to other populations with the same diagnosis)

#### Data synthesis

COSMIN guidelines for systematic reviews will be followed for the synthesis of the data extracted [34]. Data on the methods of the assessment of the loss of spinal motion caused by surgery, its measurement properties, interpretability and feasibility will be presented in an overview table for ease of interpretation. Method properties will be evaluated as per COSMIN methodology against the criteria for good measurement properties and determined as being ‘sufficient’, ‘insufficient’ or ‘indeterminate’ [34]. Following completion of the overview table, the results of each study will be compared based on the level of instrumentation. If the studies show sufficient methodological and clinical homogeneity, the results will be combined. Pooling of results will only occur where there is sufficient comparison between patient groups studied including similar age range, sex distribution, curve type and severity, similar surgical interventions made (e.g. anterior stabilisation only, posterior stabilisation only or a combination of the two) and where there is comparison between the method of measuring post-operative spinal mobility over a similar length of follow-up. From the scoping searches, these authors do not anticipate that any pooling will occur and that, consequently, a narrative synthesis of data will be reported. If appropriate, subgroup analysis can be carried out at this stage (e.g. by sex, curve severity, surgical intervention).

The recommendation of a method for the evaluation of the change in spinal motion following scoliosis surgery will depend on the properties, interpretability and feasibility of the methods described, along with an assessment of confidence in the evidence using the Grading of Recommendations Assessment, Development and Evaluation (GRADE) approach [35, 36]. As per the COSMIN guidelines, only methods with sufficient validity and at least low-quality evidence for sufficient internal consistency will be recommended [37].

## Discussion and implications of the review

The primary goals of the surgical management of AIS are to correct the spinal deformity and to prevent future progression. A side effect of this surgery, however, is a potential reduction in spinal motion post-operatively. Surgeons recognise that both AIS and its management can impact on spinal motion and therefore an individuals’ function in daily life [12, 14–17, 20]. Understanding the impact of different anatomical levels of instrumentation is crucial to clinical practice. A review looking at how differing anatomical levels of surgical fixation affect post-operative range of motion is therefore justified and this protocol aims to provide a transparent framework for a comprehensive overview and critical appraisal of the current literature. The authors expect that this work will benefit those that treat AIS, around the expected reduction in spinal motion following surgery and facilitating evidence-based discussions with patients pre-operatively. Additionally, the review will present sufficient quality evidence regarding the methods of assessing spinal motion to influence a change in the methods currently employed. 

## Supplementary Information

**Additional file 1. **Example Search Strategy Stage 1: methods of assessment of spinal motion.

**Additional file 2. **Example Search Strategy Stage 2: evaluating post-operative spinal motionbased on the level of instrumentation.

## Data Availability

The datasets used and/or analysed in this review are available from the corresponding author on reasonable request.
