# Supplementary material for: What is the most appropriate method for the measurement of the range of motion in the lumbar spine and how does surgical fixation affect the range of movement of the lumbar spine in adolescent idiopathic scoliosis? A systematic review protocol
Source: Syst Rev. 2022 Sep 30;11:208. doi: 10.1186/s13643-022-02077-1 (PMC9523991; doi:10.1186/s13643-022-02077-1)
Supplement: Supplementary file 1 — Additional file 1. Example Search Strategy Stage 1: methods of assessment of spinal motion. [file 13643_2022_2077_MOESM1_ESM.docx]

**Additional file 1**

Example Search Strategy Stage 1: methods of assessment of spinal motion

MedLine and Ovid Databases

**((((((assess*).ti,ab OR (eval*).ti,ab OR (valid*).ti,ab OR (apprais*).ti,ab OR (analy*).ti,ab OR (accura*).ti,ab) AND ((method*).ti,ab OR (technique*).ti,ab OR (way*).ti,ab)) AND ((lumbar spin*).ti,ab OR (lumbar vertebr*).ti,ab OR (back*).ti,ab OR (thoracolumbar spin*).ti,ab OR (thoracolumbar vertebr*).ti,ab)) AND ((function*).ti,ab OR (motion*).ti,ab OR (flexib*).ti,ab OR (movement*).ti,ab)) AND ((adolescent idiopathic scolios*).ti,ab OR (idiopathic scolios*).ti,ab OR (late onset scolios*).ti,ab OR (AIS).ti,ab OR (adolescent scoliosis*).ti,ab)) [Abstracts] [Document type Case Reports OR Clinical Study OR Clinical Trial OR Clinical Trial, Phase I OR Clinical Trial, Phase Ii OR Clinical Trial, Phase Iii OR Clinical Trial, Phase Iv OR Comparative Study OR Controlled Clinical Trial OR Evaluation Studies OR Meta-analysis OR Observational Study OR Randomized Controlled Trial OR Report OR Review OR Validation Studies] [Languages English] [Humans]**

EMBASE

### **((((((assess*).ti,ab OR (eval*).ti,ab OR (valid*).ti,ab OR (apprais*).ti,ab OR (analy*).ti,ab OR (accura*).ti,ab) AND ((method*).ti,ab OR (technique*).ti,ab OR (way*).ti,ab)) AND ((back*).ti,ab OR (lumbar spin*).ti,ab OR (lumbar vertebr*).ti,ab OR (thoracolumbar vertebr*).ti,ab OR (thoracolumbar spin*).ti,ab)) AND ((function*).ti,ab OR (motion*).ti,ab OR (flexib*).ti,ab OR (movement*).ti,ab)) AND ((adolescent idiopathic scoliosis*).ti,ab OR (adolescent scoliosis*).ti,ab OR (late onset scoliosis*).ti,ab OR (idiopathic scoliosis*).ti,ab OR (AIS*).ti,ab)) [Publication types Article OR Conference Paper OR Conference Proceeding OR Conference Review OR Report OR Review] [English language] [Languages English] [Human age groups School Child 7 to 12 years OR Adolescent 13 to 17 years OR Adult 18 to 64 years] [Humans]**

PubMed, Cochrane, Scopus and Web of Science Databases

**(((assess*).ti,ab OR (eval*).ti,ab OR (valid*).ti,ab OR (apprais*).ti,ab OR (analy*).ti,ab OR (accura*).ti,ab) AND ((method*).ti,ab OR (technique*).ti,ab OR (way*).ti,ab)) AND (((lumbar spin*).ti,ab OR (lumbar vertebr*).ti,ab OR (thoracolumbar spin*).ti,ab OR (thoracolumbar vertebr*).ti,ab OR (back*).ti,ab) AND ((function*).ti,ab OR (motion*).ti,ab OR (flexib*).ti,ab OR (movement*).ti,ab)) AND ((adolescent idiopathic scoliosis*).ti,ab OR (adolescent scoliosis*).ti,ab OR (late onset scoliosis*).ti,ab OR (idiopathic scoliosis*).ti,ab OR (AIS*).ti,ab))**
