# Supplementary material for: What is the most appropriate method for the measurement of the range of motion in the lumbar spine and how does surgical fixation affect the range of movement of the lumbar spine in adolescent idiopathic scoliosis? A systematic review protocol
Source: Syst Rev. 2022 Sep 30;11:208. doi: 10.1186/s13643-022-02077-1 (PMC9523991; doi:10.1186/s13643-022-02077-1)
Supplement: Supplementary file 2 — Additional file 2. Example Search Strategy Stage 2: evaluating post-operative spinal motionbased on the level of instrumentation. [file 13643_2022_2077_MOESM2_ESM.docx]

**Additional file 2**

Example Search Strategy Stage 2: evaluating post-operative spinal motion based on the level of instrumentation

MedLine and Ovid Databases

**((((adolescent idiopathic scoliosis).ti,ab OR (adolescent scoliosis*).ti,ab OR (idiopathic scoliosis*).ti,ab OR (late onset scoliosis*).ti,ab) AND ((surg*).ti,ab OR (stab*).ti,ab OR (instrument*).ti,ab OR (fusion*).ti,ab)) AND ((motion).ti,ab OR (mobility).ti,ab OR (flexib*).ti,ab OR (kinematic*).ti,ab)) [Document type Case Reports OR Clinical Study OR Clinical Trial OR Clinical Trial, Phase I OR Clinical Trial, Phase Ii OR Clinical Trial, Phase Iii OR Clinical Trial, Phase Iv OR Comparative Study OR Controlled Clinical Trial OR Evaluation Studies OR Meta-analysis OR Observational Study OR Randomized Controlled Trial OR Report OR Review OR Validation Studies] [Human age groups Child OR Adolescent OR Young adult] [Languages English] [Humans]**

EMBASE

**((((adolescent idiopathic scoliosis).ti,ab OR (adolescent scoliosis*).ti, ab OR (idiopathic scoliosis*).ti,ab OR (late onset scoliosis*).ti,ab) AND ((surg*).ti,ab OR (stab*).ti,ab OR (fusion*).ti,ab OR (instrument*).ti,ab)) AND ((motion).ti,ab OR (mobility).ti,ab OR (flexib*).ti,ab OR (kinematic*).ti,ab)) [English language] [Languages English] [Human age groups School Child 7 to 12 years OR Adolescent 13 to 17 years OR Adult 18 to 64 years] [Humans]**

PubMed, Scopus, Web of Science and Cochrane Databases

**(((adolescent idiopathic scoliosis).ti,ab OR (adolescent scoliosis*).ti,ab OR (idiopathic scoliosis*).ti,ab OR (late onset scoliosis*).ti,ab) AND ((surg*).ti,ab OR (stab*).ti,ab OR (fusion*).ti,ab OR (instrument*).ti,ab)) AND ((motion).ti,ab OR (mobil*).ti,ab OR (flexib*).ti,ab OR (kinematic*).ti,ab)**
